# Adsorptive–Photocatalytic Performance for Antibiotic and Personal Care Product Using Cu_0.5_Mn_0.5_Fe_2_O_4_

**DOI:** 10.3390/antibiotics12071151

**Published:** 2023-07-05

**Authors:** Chanat Chokejaroenrat, Chainarong Sakulthaew, Athaphon Angkaew, Apiladda Pattanateeradetch, Wuttinun Raksajit, Kanokwan Teingtham, Piyaporn Phansak, Pawee Klongvessa, Daniel D. Snow, Clifford E. Harris, Steve D. Comfort

**Affiliations:** 1 Department of Environmental Technology and Management, Faculty of Environment, Kasetsart University, Bangkok 10900, Thailand; eccnc@ku.ac.th (C.C.); athaphon.ak@gmail.com (A.A.); apiladda.pat@ku.th (A.P.); ecpwk@ku.ac.th (P.K.); 2 Department of Veterinary Technology, Faculty of Veterinary Technology, Kasetsart University, Bangkok 10900, Thailand; cvtwnr@ku.ac.th; 3 Department of Agronomy, Faculty of Agriculture at Kamphaeng Saen, Kasetsart University, Nakhon Pathom 73140, Thailand; agrknw@ku.ac.th; 4 Division of Biology, Faculty of Science, Nakhon Phanom University, Nakhon Phanom 48000, Thailand; pphansak@npu.ac.th; 5 Water Sciences Laboratory, University of Nebraska-Lincoln, Lincoln, NE 68583, USA; dsnow1@unl.edu; 6 Department of Chemistry and Biochemistry, Albion College, Albion, MI 49224, USA; charris@albion.edu; 7 School of Natural Resources, University of Nebraska-Lincoln, Lincoln, NE 68583, USA; scomfort1@unl.edu

**Keywords:** adsorption–photocatalysis integration, adsorption isotherms, adsorption kinetics, Cu_0.5_Mn_0.5_Fe_2_O_4_ nanoparticles, oxytetracycline removal, paraben removal, root anatomical changes, seed germination

## Abstract

The amount of antibiotics and personal care products entering local sewage systems and ultimately natural waters is increasing and raising concerns about long-term human health effects. We developed an adsorptive photocatalyst, Cu_0.5_Mn_0.5_Fe_2_O_4_ nanoparticles, utilizing co-precipitation and calcination with melamine, and quantified its efficacy in removing paraben and oxytetracycline (OTC). During melamine calcination, Cu_0.5_Mn_0.5_Fe_2_O_4_ recrystallized, improving material crystallinity and purity for the adsorptive–photocatalytic reaction. Kinetic experiments showed that all four parabens and OTC were removed within 120 and 45 min. We found that contaminant adsorption and reaction with active radicals occurred almost simultaneously with the photocatalyst. OTC adsorption could be adequately described by the Brouers–Sotolongo kinetic and Freundlich isotherm models. OTC photocatalytic degradation started with a series of reactions at different carbon locations (i.e., decarboxamidation, deamination, dehydroxylation, demethylation, and tautomerization). Further toxicity testing showed that *Zea mays* L. and *Vigna radiata* L. shoot indexes were less affected by treated water than root indexes. The *Zea mays* L. endodermis thickness and area decreased considerably after exposure to the 25% (*v*/*v*)-treated water. Overall, Cu_0.5_Mn_0.5_Fe_2_O_4_ nanoparticles exhibit a remarkable adsorptive–photocatalytic performance for the degradation of tested antibiotics and personal care products.

## 1. Introduction

Pharmaceuticals and personal care products (PPCPs) are substances used for medical, cosmetics, hygiene, and health care. The increased global production of PPCPs and subsequent disposal without environmental controls have negatively impacted some soil–water environments. While newly developed personal care products are continuously entering the environment, older, previously disposed PPCPs may also be a concern. Many PPCPs are non-biodegradable in aerobic environments, especially nitrogen-containing compounds [1]. Because natural PPCP degradation can take several months to years, both parent structure and degradation products can still be found in the environment, long after their initial discharge [2]. Some PPCPs have the capability to be transferred from disposal streams (i.e., manure amendments, wastewater irrigation, and sludge disposal) to agricultural lands, crops, and then humans or animal dietary intake [3]. Because of this, human blood, urine, and breast milk have all recently been found to contain some PPCPs [4,5].

The detection of PPCPs in animals and humans confirms that conventional wastewater treatment approaches are inadequate. Moreover, trace concentrations of PPCPs can produce potential adverse effects on human health, aquatic animals, and aquatic ecosystems [6]. Consequently, there is a need for novel water treatment that can effectively reduce PPCPs while simultaneously removing traditionally encountered organic contaminants. Advanced oxidation processes (AOPs) are primarily recognized for their strong reactivity and harmless by-product production [7]. Heterogeneous photo-Fenton-like processes are considered AOPs and have been previously used in several PPCP treatment [8,9]. 

The selection of freshly synthesized materials that are reusable and capable of operating across a broad pH range is vital to the success of remedial technologies. By employing manganese ferrite (MnFe_2_O_4_) as heterogeneous catalysts, researchers have found it to be more effective than other catalysts. This is due to the various valences in the nanocomposites, including Mn^4+^, Mn^3+^, Mn^2+^, Fe^3+^, and Fe^2+^, which can trigger the synergistic action between the Mn and Fe redox cycles [10,11]. Furthermore, doping manganese ferrite structures with another transition metal (e.g., Cu) can produce ternary transition metal oxides (e.g., Cu_0.5_Mn_0.5_Fe_2_O_4_), which have a greater surface area than iron-based transition metal catalysts [12,13]. This is beneficial to photo-Fenton-like AOP treatments by providing larger active sites, an excellent oxygen exchangeability, and an outstanding capability for electron transfer through the Cu^2+^/Cu^1+^ redox cycle. These characteristics facilitate activating hydrogen peroxide (H_2_O_2_) and producing hydroxyl radicals that enhance the degradation of recalcitrant organic pollutants [12,13]. As such, Cu_0.5_Mn_0.5_Fe_2_O_4_ had higher catalytic activity than other single spinel ferrites (e.g., CuFe_2_O_4_, MnFe_2_O_4,_ etc.) in activating H_2_O_2_ under ultraviolet (UV) light and was unlikely to cause secondary pollution from metal leaching due to its high stability [14,15]. In addition, several researchers reported that Cu_0.5_Mn_0.5_Fe_2_O_4_ nanoparticles could be successfully used as an adsorbent to remove several contaminants from wastewater [12,14,16]. However, an evaluation of the cooperative impact between the adsorption and photocatalysis of Cu_0.5_Mn_0.5_Fe_2_O_4_ nanoparticles for removing PPCPs has never been reported.

In this study, we selected two representatives for PPCPs: (1) oxytetracycline (OTC), an antibiotic generally used in aquatic veterinary practice, and (2) parabens (e.g., methyl-, ethyl-, propyl-, and butylparaben), an antimicrobial preservative often used in cosmetic and care products. Our objective was to determine the adsorptive–photocatalytic degradation performance of OTC and parabens using Cu_0.5_Mn_0.5_Fe_2_O_4_ synthesized using a simple co-precipitation method. The physicochemical properties of Cu_0.5_Mn_0.5_Fe_2_O_4_ before and after use were characterized. In the end, we determined any residual toxicity impacts of the treated water on seed germination, seedling growth, and anatomical root changes for *Zea mays* L. and *Vigna radiata* L.

## 2. Materials and Methods

### 2.1. Chemicals

Analytical-grade substances from different sources and deionized water (DI) were employed in this research. Loba Chemie Pvt. Ltd. (Mumbai, India) supplied manganese sulfate monohydrate (MnSO_4_·H_2_O, 99%). QRëC (Auckland, New Zealand) supplied ferric chloride hexahydrate (FeCl_3_∙6H_2_O, >98%) and acetic acid (CH_3_COOH, 99.8%). Merck (Darmstadt, Germany) supplied sodium hydroxide (NaOH), hydrogen peroxide (30%, H_2_O_2_), copper sulfate (CuSO_4_, 99%), oxytetracycline dihydrate (C_22_H_24_N_2_O_9_·2H_2_O; OTC, ≥99%), methyl 4-hydroxybenzoate (C_8_H_8_O_3_; methylparaben, MP, ≥99%), ethyl 4-hydroxybenzoate (C_9_H_10_O_3_; ethylparaben, EP, ≥99%), propyl 4-hydroxybenzoate (C_10_H_12_O_3_; propylparaben, PP, ≥99%), and butyl 4-hydroxybenzoate (C_11_H_14_O_3_; butylparaben, BP, ≥99%). RCI Labscan (Bangkok, Thailand) supplied acetonitrile (C_2_H_3_N; ACN) and methanol (CH_3_OH; MeOH). Alfa Aesar (Shanghai, China) supplied melamine (C_3_H_6_N_6_, 99%).

### 2.2. Synthesis of Cu_0.5_Mn_0.5_Fe_2_O_4_ Nanoparticles

The Cu_0.5_Mn_0.5_Fe_2_O_4_ nanoparticles were synthesized via co-precipitation and melamine-assisted calcination. We mixed 125 mL of CuSO_4_ (0.2 M), 125 mL of MnSO_4_·H_2_O (0.2 M), 250 mL of FeCl_3_∙6H_2_O (0.4 M), and 500 mL of DI at 80 °C for 1 h. To raise the pH to 10.5, we gently dripped 8 M NaOH solution into the mixture for 1 h. After stirring for another hour, we filtered the precipitates by applying vacuum, washed them with DI water and ethanol multiple times, and dried them at 95 °C for 15 h. The dry particles were blended with melamine in a crucible at a ratio of 2:1. The resulting mixture was subjected to calcination at a temperature of 550 °C for a duration of 3 h, resulting in the production of the final product.

### 2.3. Chemical and Material Analyses

Temporal changes in OTC and paraben concentrations were analyzed by high-performance liquid chromatography (HPLC) using a photodiode array detector (Waters). With a 20 μL injection volume, an isocratic mobile phase of acetonitrile and 0.1% (*v*/*v*) acetic acid (20:80) was used for OTC analysis, whereas a mobile phase of acetonitrile and DI (60:40) was used for paraben analysis. Using a flow rate of 1 mL min^−1^, samples were separated by a Reversed-Phase 18 (RP-18) Mightysil HPLC column (250 × Ø 4.6 mm) connected with a guard column. Sample peaks were quantified at 354 nm wavelength for OTC and 254 nm for four types of parabens (i.e., methyl-, ethyl-, propyl-, and butylparaben, or MP, EP, PP, and BP) using an external calibration curve.

Morphological structures were analyzed using JEOL (JSM-6010) scanning electron microscopy and Talos (F200X) high-resolution transmission electron microscopy (HRTEM). Material crystallinity characteristics were obtained from a D2 Phaser Bruker X-ray diffractor (XRD), whereas the surface functional groups were acquired from a Bruker (Tensor 27) Fourier transform infrared spectroscopy (FTIR).

Following the photocatalytic degradation experiments, we used ProElut C18 solid-phase extraction cartridges (Dikma) to concentrate the OTC degradates prior to further analyzing them on a liquid chromatography mass spectrophotometer (LC/MS). Initially, the cartridge was preconditioned with 5 mL of methanol followed by 5 mL of ultrapure water at a flow rate of 2 mL min^−1^. A total of 60 mL of samples was introduced into the cartridge at a flow rate of 5 mL min^−1^. Then, the samples were eluted with 2 mL of acetonitrile into the glass test tubes prior to filtrating with a 0.45 μm polytetrafluoroethylene (PTFE) syringe filter and transferred to vials for LC/MS analysis (Agilent 6420).

An isocratic mobile phase of freshly prepared acetonitrile and 0.1% (*v*/*v*) acetic acid (20:80) was used with a 10 μL injection volume. Using a flow rate of 0.2 mL min^−1^, samples were separated by a Mightysil C18 column (250 × Ø 4.6 mm). Mass spectral data were obtained by scanning the quadrupole from 200 to 500 m/z with a 1 sec scan and a 30 V cone voltage setting with the following conditions: electrospray ionization source (positive ion mode), 3.7 kV electrospray voltage, 75 psi nebulization gas pressure, 500 °C heater temperature, and 400 °C capillary temperature.

### 2.4. Varying Dosage of H_2_O_2_ or Catalysts

Two independent variables that can affect the photo-Fenton catalytic performance are H_2_O_2_ and catalyst dosage. Here, we varied both parameters (H_2_O_2_: 0.1–0.6 mg L^−1^, Cu_0.5_Mn_0.5_Fe_2_O_4_: 0–0.5 g L^−1^) and determined the temporal changes of OTC and paraben concentrations. Initially, 100 mL of 0.1 mM OTC was placed in a 250 mL beaker with a designated catalyst and stirred in the absence of light for 30 min to reach adsorption equilibrium. Then, the beaker was irradiated with a simulated sunlight source using a commercial 75-watt halogen lamp. At a preselected time, 1 mL of sample was filtered through a 0.45 µm PTFE syringe filter. To stop the reaction, 0.7 mL of filtrated sample was transferred to an HPLC vial containing 0.875 mL of methanol. Samples were then stored in a refrigerator at 4 °C until HPLC analysis.

### 2.5. OTC Adsorption Study

#### 2.5.1. Adsorption Kinetics

The OTC solution was freshly prepared before use in the kinetic and isotherm experiments. A total of 30 mL of 0.1 mM OTC concentrations was placed in a 40 mL amber vial. We selected 6 mg, 12 mg, and 18 mg of Cu_0.5_Mn_0.5_Fe_2_O_4_ as adsorbents in this kinetic study (equivalent to 0.2, 0.4, and 0.6 g L^−1^). The experiments were performed in quadruplicate. The experiment started once the adsorbent had been added. Vials were shaken on a reciprocating shaker at 200 rpm. A total of 0.75 mL of samples was collected at 0.5, 1, 2, 3, 4, 5, and 6 h and filtrated using a 0.45 μM PTFE syringe filter. Samples were kept at 4 °C until HPLC analysis.

The kinetic models used in this study were pseudo first-order, pseudo second-order, Elovich, Brouers–Sotolongo (order 2), and intra-particle diffusion (2 phases) kinetic models (Table 1). The parameters in these models were determined by least squares approximation. In other words, the model used the parameter values that yielded the lowest sum of squared error (SSE) between observed and modeled values (Equation (1)).

(1) $$SSE=\sum_{i=1}^{n}{\left(y_{i}-\hat{y_{i}}\right)}^{2}$$

where n is the count of data, $y_{i}$ is the value of i the observed data (observed amount of adsorbed OTC), and $\hat{y_{i}}$ is the value of i modelled data (calculated amount of adsorbed OTC). For each kinetic model, the values of parameters that gave the lowest SSE were determined by a generalized reduced gradient (GRG) algorithm [17] with multiple starting points. The GRG algorithm was performed by the Solver add-in in Microsoft Excel.

#### 2.5.2. Adsorption Isotherms

Three amounts of Cu_0.5_Mn_0.5_Fe_2_O_4_ (6, 12, and 18 mg) were selected for the adsorption isotherm study (equivalent to 0.2, 0.4, and 0.6 g L^−1^). The OTC concentrations, which ranged from 0.005, 0.01, 0.025, 0.05, 0.75, 0.1, 0.15, and 0.2 mM, were placed in each vial. The experimental set-up and sample collecting protocol were similar to the adsorption kinetic experiment.

#### 2.5.3. Isotherm Models

This study employed multiple isotherm models (Table 1). However, in this study, the values of parameters cannot be clearly determined for some models. Only the models that give clear values of parameters were selectively reported. These models include Langmuir, Freundlich, and Temkin isotherm models. Since these models can be transformed into linear forms (Equations (2)–(4) for Langmuir, Freundlich, and Temkin isotherm models, respectively), the parameters in these models were determined by least squares linear regression.

(2) $$\frac{1}{q_{e}}=\frac{1}{q_{m}b}\left(\frac{1}{C_{e}}\right)+\frac{1}{q_{m}}$$

(3) $${ln\;q}_{e}=\frac{1}{n}\left(ln{\;C}_{e}\right)+ln{\;K}_{f}$$

(4) $$q_{e}=\frac{RT}{B_{T}}\left(lnC_{e}\right)+\frac{RT}{B_{T}}\left(lnA_{T}\right)$$

From the linearized Langmuir isotherm model (Equation (2)), the value of $1/\left(q_{m}b\right)$ was the slope of the linear relationship between $1/C_{e}$ (independent variable) and $1/q_{e}$ (dependent variable), and the value of $1/q_{m}$ was the y-intercept of that linear relationship. By using a similar approach, the values of $K_{f}$ and n in the Freundlich isotherm model (Equation (3)) and the values of $A_{T}$ and $B_{T}$ in the Temkin isotherm model (Equation (4)) could be determined. 

#### 2.5.4. Model Evaluation

In this study, the kinetic and isotherm models were evaluated by root mean square error (RMSE) and coefficient of determination ($R^{2}$) (Equations (5) and (6)).

(5) $$RMSE=\sqrt{\frac{1}{n}\sum_{i=1}^{n}{\left(y_{i}-\hat{y_{i}}\right)}^{2}}$$

(6) $$R^{2}=1-\frac{\sum_{i=1}^{n}{\left(y_{i}-\hat{y_{i}}\right)}^{2}}{\sum_{i=1}^{n}{\left(y_{i}-\bar{y}\right)}^{2}}$$

where RMSE is the root mean square error, n is the count of data, $y_{i}$ is the value of i th observed data, $\hat{y_{i}}$ is the value of i th modelled data, $R^{2}$ is the coefficient of determination, and $\bar{y}$ is the average value of the observed data. A low RMSE and high $R^{2}$ indicate that the model is suitable. Conversely, a high RMSE and low $R^{2}$ indicate that the model is not suitable [20].

Since the isotherm models were parameterized by least squares linear regression, the strengths of the linear relationships were also measured using squared Pearson correlation coefficients ($r^{2}$) (Equation (7)). Moreover, the adjustment of $r^{2}$ according to the count of predictor(s) was also performed (Equation (8)) [21].

(7) $$r^{2}=\frac{{\left(\sum_{i=1}^{n}\left(x_{i}-\bar{x}\right)\left(y_{i}-\bar{y}\right)\right)}^{2}}{\sum_{i=1}^{n}{\left(x_{i}-\bar{x}\right)}^{2}\sum_{i=1}^{n}{\left(y_{i}-\bar{y}\right)}^{2}}$$

(8) $$r_{adj}^{2}=1-\frac{\left(1-r^{2}\right)\left(n-1\right)}{\left(n-p-1\right)}$$

where $r^{2}$ is the squared Pearson correlation coefficient, n is the count of data, $x_{i}$ is the value of i th independent variable, $\bar{x}$ is the average value of the independent variable, $y_{i}$ is the value of i th dependent variable, $\bar{y}$ is the average value of the dependent variable, $r_{adj}^{2}$ is the $r^{2}$ adjusted according to the count of predictor(s), and p is the count of predictor(s).

### 2.6. Evaluating Effects of Treated Water on Seed Germination and Root Anatomy

Due to their prevalence in Thai agriculture, *Zea mays* L. and *Vigna radiata* L. were used as representative species (monocotyledon vs. dicotyledon) for assessing the effects of treated water on seedling development and root morphological features. *Zea mays* L. seeds (cv.SW5720, National Corn and Sorghum Research Center, Nakhon Ratchasima, Thailand) and *Vigna radiata* L. seeds (cv.KUML4, Department of Agronomy, Faculty of Agriculture at Kamphaeng Saen, Kasetsart University, Nakhon Pathom, Thailand) were selected for this purpose. The OTC-untreated and treated water were assigned in two separate studies with varying percentages of OTC solution (5 to 15% and 25 to 50%). Fifty seeds from each treatment were planted in a box (19 × 28 × 11 cm) containing 2.55 kg of sand and 450 mL of three previously described waters and incubated at 25 ºC. Each treatment was repeated four times and arranged in a completely random design (CRD). In order to evaluate the vigor and physiological performance of the seeds, the length of the shoots (the length from ground level to the tip of the longest leaf) and roots (the length from the base of the stem to the end of the longest root) were measured 7 d after planting (*Zea mays* L.) or 8 d (*Vigna radiata* L.). Normal seedlings’ shoots and roots were weighed in mg (per plant) after drying in a hot-air oven at 80 °C for 24 h. All the obtained data were statistically evaluated. The mean germination time (MGT) and vigor index (VI) were calculated using the following formulas (Equations (9) and (10)):

(9) $$MGT(days)=\frac{\sum \left(n*d\right)}{N}$$

(10) $$VI=Final\;germination\left(\%\right)\times Seedling\;dry\;weight$$

where n is number of seeds germinated on each day, d is number of days from germination, and N is total number of germinated seeds.

Because *Zea mays* L. and *Vigna radiata* L. roots exhibited varying degrees of sensitivity to the constituents present in the treated water, leading to alterations in key anatomical features, their anatomical studies provided valuable insights into the potential physiological and developmental impacts of treated water on root tissues. We investigated the effect of treated water on root anatomy, focusing on parameters such as endodermis thickness, endodermis area, vascular cylinder diameter, metaxylem area, and cortex. These anatomical parameters are responsible for water and nutrient uptake, anchoring the plant, and providing structural support. Three roots per treatment per replication were anatomically analyzed.

The roots were immersed for 2 days in the FAA fixative solution (i.e., formalin (10%)/glacial acetic acid (5%)/ethanol (50%)/DI water (35%)). A table microtome and free-hand sectioning were used to generate cross sections at 4 ± 0.5 cm from the root apex to study *Zea mays* L. and *Vigna radiata* L. root anatomy. The slices were dyed with a 1% safranin-O dye solution for 2 min, mounted on glass slides with 50% glycerin, and then inspected and photographed using a light microscope coupled with Zen 3.5 software (Carl Zeiss, Axio lmage 2, Oberkochen, Germany). Tukey’s honest significant difference (HSD) was used to compare treatment means at *p* = 0.05.

## 3. Results and Discussion

### 3.1. Cu_0.5_Mn_0.5_Fe_2_O_4_ Characteristics

**Figure 1:**
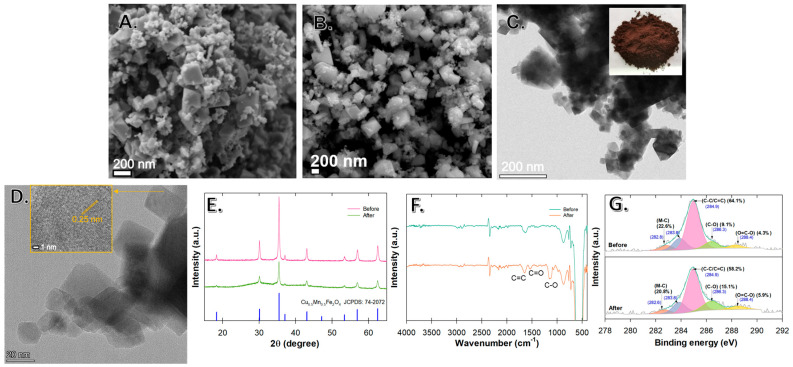
Cu_0.5_Mn_0.5_Fe_2_O_4_ nanoparticle characteristics: (**A**,**B**) SEM images; (**C**) TEM image with a visual observation of particles; (**D**) HRTEM image. The nanoparticle before and after use in photocatalytic reaction: (**E**) XRD patterns; (**F**) FTIR spectra; and (**G**) C 1s XPS spectra.

The SEM images of Cu_0.5_Mn_0.5_Fe_2_O_4_ exhibited a rough, uneven, and porous network structure caused by smaller particles that were well-distributed among larger particles, evidencing the flawless melamine-assisted calcination (Figure 1A,B). The TEM images depict the arrangement of large octahedral shapes surrounded by smaller particle sizes (Figure 1C,D). It is obvious that the morphological characteristics of the material were consistent with those observed through SEM. The HRTEM images also showed lattice fringes of 0.25 nm corresponding to the (311) plane (Figure 1D). In addition, the magnetic properties were more pronounced in comparison to the catalyst that was prepared without melamine addition. This was attributed to the recrystallization process that occurred during the calcination stage with melamine, which served as a coordinating agent. It was confirmed that melamine provided a platform and coordinated with the co-precipitated particles during calcination, resulting in promoting the Cu_0.5_Mn_0.5_Fe_2_O_4_ formation without impurity and providing a carbon source for the M-C and C-C/C=C bonds forming a heterojunction structure with Cu_0.5_Mn_0.5_Fe_2_O_4_ [12] (Figure 1E–G). Like carbon-based material doping, these carbons could act as an electron acceptor by suppressing photo-excited electron–hole recombination and enhancing the light absorption capability, thereby improving Cu_0.5_Mn_0.5_Fe_2_O_4_ photocatalytic activity [22,23]. This ultimately facilitated the formation of a ferrite structure without the α-Fe_2_O_3_ impurities [12]. 

While the XRD spectra of both materials (before and after use in adsorptive–photocatalytic activity) show similar diffraction peaks at 2θ values of 18.3°, 30.0°, 35.4°, 37.0°, 43.0°, 53.3°, 56.8°, and 62.4° corresponding to the diffraction crystal planes (111), (220), (311), (222), (400), (422), (511), and (440), the unused materials had a slightly higher peak intensity (Figure 1E). This indicates that the occupying of adsorption sites may have occurred, but the crystallinity was still intact, which would still be able to provide a great photocatalytic performance. 

The strong vibration spectra in the low wavenumber region (450 to 700 cm^−1^) were assigned to the M–O band (M=Cu, Mn, and Fe) (Figure 1F). These bands confirmed the formation of M–O bonds at octahedral sites (Cu^2+^–O^2−^, and Mn^2+^–O^2−^ stretching vibration) and tetrahedral sites (Fe^3+^–O^2−^ stretching vibration) of the Cu_0.5_Mn_0.5_Fe_2_O_4_ nanocomposite surface [13,24,25,26]. These surface metals play an important role in heterogeneous catalytic reactions. The peaks at 1150, 1530, 1640, and ~3000 cm^−1^ can be attributed to C-O, C=O, C=C, and sp2 (–CH) or sp3 (=CH) hybridized carbon atoms (Figure 1F) [25,27,28]. The XPS analysis of the C 1s spectra indicates that the peaks at 282.8 and 283.8 eV corresponded to M-C bonds, while the other peaks at 284.9, 286.3, and 288.4 eV were attributed to C-C/C=C, C-O, and O=C-O (Figure 1G) [25,29]. It also reveals that, after use, C-C and C=C were decreased, while C-O and C=O were increased, indicating that the surface carbons undergo partial oxidation by the active oxygen species or exhibit involvement in the catalytic reaction.

Furthermore, despite the fact that the addition of melamine would result in C–N containing peaks that would aid in the crystal reformation, we did not see such peaks in XRD, FTIR, or XPS analyses. This may be explained by the role of melamine during the calcination stage. Upon heating melamine with the co-precipitated particles, a platform for the distribution of nanoparticles was created. This process was coordinated with the transition metals, leading to the formation of a metal–melamine complex [30,31]. At a high calcination temperature of 550 °C, this compound can undergo further decomposition, resulting in the formation of a spinel ferrite structure and the release of volatile gases (i.e., CO_X_, NO_X_, and NH_3_) [32]. The findings validate the function of melamine as a coordinating agent that effectively enhances the crystalline structure of Cu_0.5_Mn_0.5_Fe_2_O_4_ and successfully eliminates the presence of Fe_2_O_3_ impurities.

### 3.2. Photocatalytic Performance

#### 3.2.1. Paraben Degradation Efficiency

**Figure 2:**
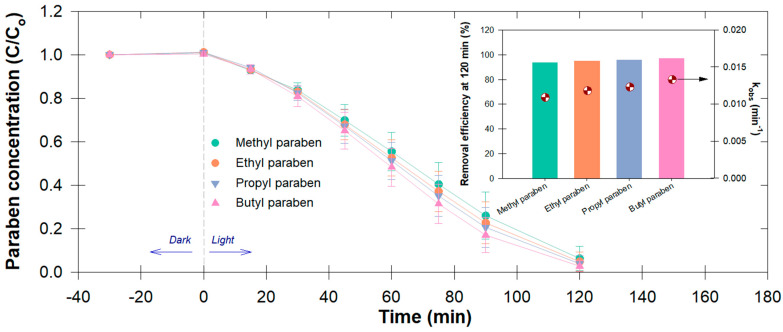
Temporal changes in paraben concentration (i.e., methyl-, ethyl-, propyl-, and butylparaben, or MP, EP, PP, and BP) under the adsorptive–photocatalysis process; (inset graph) removal efficiency (at 120 min) and observed degradation rates of parabens (*k_obs_*; min^−1^).

A photocatalytic experiment revealed that a >95% paraben degradation efficiency was obtained in 120 min (Figure 2). We did not observe a significant change in the first 30 min in the dark, indicating that the adsorption–desorption equilibrium of catalysts had been reached. The insignificant difference between parabens in the first 30 min of light-irradiation was probably due to the insufficient energy for initiating active oxygen species production. The temporal monitoring of each paraben concentration revealed a rise in the observed pseudo first-order rate constant (*k_obs_*), and %removal at 120 min in the order BP > PP > EP > MP (inset of Figure 2), which was due to the generated ^•^OH, photoelectron (e^−^), and ^•^O_2_^−^, confirmed by our previously proposed radical formation mechanisms in our recent publication by Angkaew et al. [12], which was similarly observed by other works [33,34]. The BP highest degradation efficiency was due to the BP molecular structure with a longer ester chain and the existence of more unsaturated C=C bonds, both of which were preferentially targeted by ^•^OH and ^•^O_2_^−^ [35,36]. These results validate the occurrence of the reaction with ^•^OH in a correlation with the alkyl chain length of the paraben [37].

**Figure 3:**
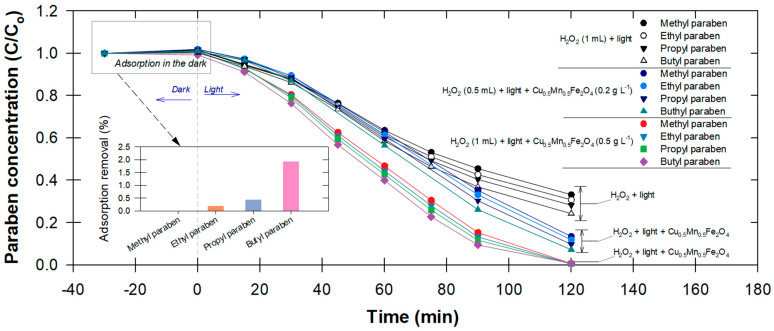
Temporal changes in paraben concentration (i.e., methyl-, ethyl-, propyl-, and butylparaben) under three different oxidative conditions; (inset graph) adsorption removal of each paraben in the absence of light irradiation.

When the photocatalytic activity was tested by removing parabens individually at different H_2_O_2_ concentrations and Cu_0.5_Mn_0.5_Fe_2_O_4_ dosages, the BP degradation rates were again the fastest, confirming that its molecular structure is more susceptible to oxidative species (Figure 3). The degradation rate exhibited a corresponding increase with the increase in catalyst doses. The degradation rate did not, however, rise by 2.5 times as expected in response to the increase in catalyst dosage, but rather by 1.25 times. Nevertheless, the larger specific area was responsible for this increase because it provided more adsorptive and reactive sites for the H_2_O_2_ that was present, accelerating the production of reactive species (Figure 3).

It is noted that, in the dark, BP was also adsorbed, even though there was no photoactivity involved (Figure 3). Up to a 40% increase in paraben removal efficiency was observed following an increase in the Cu_0.5_Mn_0.5_Fe_2_O_4_ dose to 0.5 g L^−1^. This proves that, besides photocatalytic activity, the availability of adsorption sites and a contact surface area also increased. However, Hashemian, et al. [16] reported that, when the quantity of adsorbent exceeds 1.0 g, the increase in removal of pollutants can become negligible due to the occurrence of particle–particle interactions, such as aggregation, which subsequently diminish the available surface area for adsorption sites, and that the adsorption efficiency may play a synergistic important role in this oxidative system.

#### 3.2.2. Oxytetracycline (OTC) Degradation Efficiency

**Figure 4:**
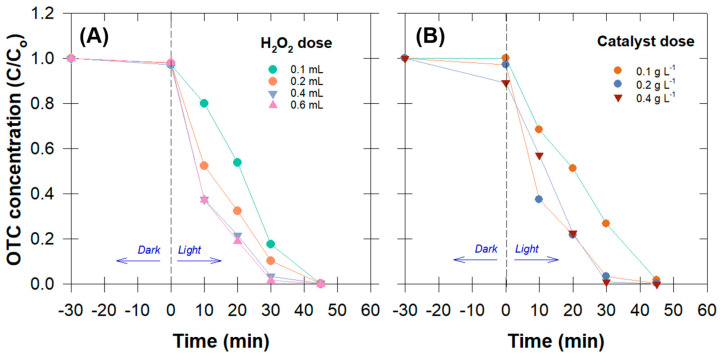
Temporal changes in oxytetracycline concentration (OTC) under the adsorptive–photocatalysis process at varied concentrations of H_2_O_2_ (**A**) and catalyst dose (**B**).

The results of lowering H_2_O_2_ and catalyst dosages more than the previous experiment show that the increase in these dosages did not necessarily increase the OTC degradation efficiency (Figure 4). The increase in H_2_O_2_ resulted in a better photocatalytic activity, except for the H_2_O_2_ doses of 0.4 mL and 0.6 mL, which had a similar degradation efficiency (Figure 4A). This could have been a lack of active sites for the catalyst dose to initiate the reaction. One other possible explanation is that the created ^•^OH was quenched by the self-scavenging action of the abundant H_2_O_2_, which itself deteriorated into H_2_O and O_2_ (Equations (11)–(13)) [10,29].

H_2_O_2_ + H_2_O_2_ → H_2_O + O_2_ (11)

H_2_O_2_ + ^•^OH → HO_2_^•^ + H_2_O (12)

HO_2_^•^ + ^•^OH → O_2_ + H_2_O (13)

Increases in the Cu_0.5_Mn_0.5_Fe_2_O_4_ dosage had a similar effect on OTC degradation efficiency as increases in the H_2_O_2_ concentration did (Figure 4B). Besides the lack of H_2_O_2_ concentration for initiating the reaction, a self-scavenging effect may have occurred from the excessive amounts of Cu^+^, Fe^2+^, and Mn^2+^ in the solution (Equations (14)–(16)) [25,38].

Cu^+^ + ^•^OH → Cu^2+^ + OH^−^ (14)

Mn^2+^ + ^•^OH → Mn^3+^ + OH^−^ (15)

Fe^2+^ + ^•^OH → Fe^3+^ + OH^−^ (16)

Moreover, an excessive amount of suspended catalysts might prevent light from reaching the reaction sites, reducing the amount of ^•^OH produced. Notably, the removal efficiency anomaly (>10%) in the dark was observed for the highest catalyst dose (0.4 g L^−1^) (Figure 4B). This phenomenon indicates the existence of catalyst adsorptivity prior to the generation of active radicals and that some contaminants may have been adsorbed on the catalyst surface. As a result, we ruled out the idea that adsorption was also a key factor in pollution removal. This information is valuable as it pertains to the potential utilization of greater quantities of catalyst in practical applications.

### 3.3. Adsorptive Performance

At the beginning of the adsorption experiment, a high adsorption rate was seen for all three chosen amounts. This indicates that the catalyst was a viable adsorbent and that there were plenty of adsorption sites accessible, especially as the adsorbent amount increased. This might be attributed to our selected synthesis approaches that used melamine-assisted calcination, which resulted in its octahedral morphology surrounded by tiny spheres. Thus, this is advantageous for both excellent adsorption and excellent photocatalytic properties.

In this study, pseudo first-order, pseudo second-order, Elovich, and Brouers–Sotolongo models were used to explain adsorption, and two-phase intra-particle diffusion was used to explain diffusion (Table 1). RMSE and $R^{2}$ values were used to determine the best fit model.

**Figure 5:**
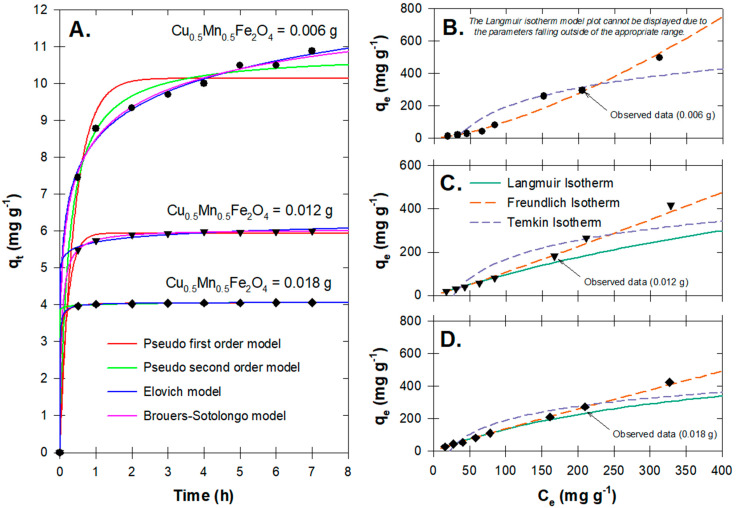
OTC adsorption on Cu_0.5_Mn_0.5_Fe_2_O_4_ nanoparticles; (**A**) observed and modeled kinetic profiles; and (**B**–**D**) observed and modeled equilibrium isotherm profiles.

Figure 5A shows the plot of the fitted adsorption models (pseudo first-order, pseudo second-order, Elovich, and Brouers–Sotolongo models). Differences among results from these models can be seen when the Cu_0.5_Mn_0.5_Fe_2_O_4_ amount is not very high. Overall, both the Brouers–Sotolongo and the pseudo second-order kinetic models described the OTC-Cu_0.5_Mn_0.5_Fe_2_O_4_ adsorption kinetics well, as indicated by the low RMSE and high $R^{2}$ (Table 2). Therefore, it can be concluded that the adsorption process between the adsorbent and OTC molecules in the liquid phase was a chemisorption process, and that the efficiency was entirely dependent on the amount of adsorbent used and the concentration of the adsorbate. The OTC-adsorbed mass per Cu_0.5_Mn_0.5_Fe_2_O_4_ mass at the equilibrium ($q_{e}$) was highest for the lowest Cu_0.5_Mn_0.5_Fe_2_O_4_ amount (i.e., 0.006 g) and the decrease at higher Cu_0.5_Mn_0.5_Fe_2_O_4_ amounts was due to their more available adsorption sites (Table 2; Figure 5).

At 0.006 g of Cu_0.5_Mn_0.5_Fe_2_O_4_, the Brouers–Sotolongo model shows a notably greater $q_{e}$ than the pseudo second-order model, and the accuracies of these models are low (RMSEs are high and $R^{2}$s are low), implying that the predicted data may not be as accurate at an extremely low adsorbent amount (Table 2). At higher amounts of Cu_0.5_Mn_0.5_Fe_2_O_4_, the predicted adsorbed concentrations from both models were similar (6.04 and 4.06–4.07 mg g^−1^ at 0.012 and 0.018 g of Cu_0.5_Mn_0.5_Fe_2_O_4_, respectively) and the accuracies of the models are high (RMSEs are low and $R^{2}$s are high). By comparing the values of $R^{2}$, the OTC adsorption mechanism appeared to be best described by the Brouers–Sotolongo equation. Adsorbate/adsorbent interactions were found to be fractal when the fractal time exponent (γ; also called the global fractal time) was less than 1 [39,40]. This may be attributed to the material’s powder-like properties, which may preferentially reside on the edge wall and the inner lid, decreasing the amount of contact with the OTC molecules.

With an increase in adsorbate amount, agglomeration may have started to occur, rendering the adsorbent attachment to the edge wall unlikely. As can be seen, the experiment was contaminant-specific and was conducted in the dark. By using OTC as the target contaminant, the results show that adsorption is playing the main role as OTC was tentatively adsorbed before the start of the photocatalytic performance.

Ho and McKay [41] proposed that the adsorbate diffused through the liquid phase and the film surrounding the adsorbent surface before interacting with the functional groups of the adsorbent–adsorbate (Table 2). Consequently, the kinetic data previously acquired were utilized to selectively examine the intra-particle diffusion stages.

Based on our findings, the adsorption phases (i.e., double-linearity characterization) were divided at 0.51–0.58 h, indicating that the external mass transfer followed by intraparticle diffusion may occur at even lower amounts of adsorbent. This allows OTC molecules to transport to different phases prior to reaching equilibrium. As the adsorbent dosage increased, the first-phase slopes ($K_{d}$) decreased, suggesting that self-agglomeration had slightly occurred and that the adsorbent boundary layers had thickened. The rapid disruption in the $K_{d}$ indicated prompt adsorption on the adsorbent surface, while the subsequent slower rates of adsorption (i.e., a lower $K_{d}$) confirmed the occurrence of diffusional phenomena within the adsorbent particles [42,43].

By comparing the values of RMSE and $R^{2}$ among three isotherm models (Table 1 and Table 3; Figure 5), the Freundlich model better described the adsorption isotherm data, indicating that multilayer adsorption was dominant and that the Cu secondary doping facilitated the uniformly distributed surface for OTC molecules. The n value increased to >1 at 0.018 g of adsorbent, indicating the system’s unfavorability [44]. This was probably due to the agglomeration occurring at a higher adsorbent dose, which coincided with the previously described kinetic experiments. Overall, the results provide proof that, during the adsorption/desorption equilibrium, adsorption can occur prior to the photocatalytic reaction that serves as the main oxidation mechanism for degrading unadsorbed OTC molecules.

### 3.4. OTC Degradation Products

**Figure 6:**
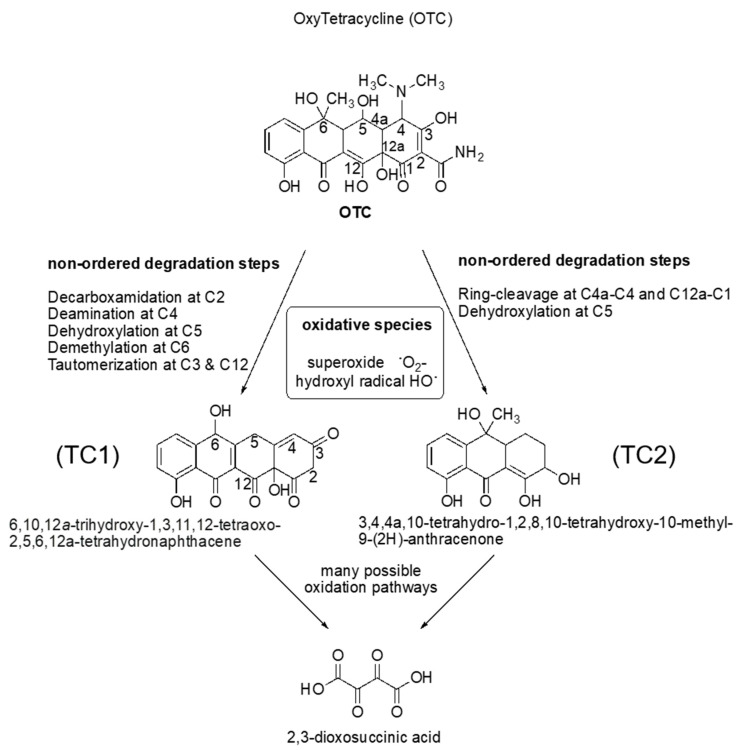
OTC degradation mechanism following the photo-Fenton catalytic activity of Cu_0.5_Mn_0.5_Fe_2_O_4_ nanoparticles.

Almost certainly, multiple mechanisms for the oxidative degradation of OTC (m/z = 460) are occurring simultaneously in this system. The identified intermediates allow us to suggest the reactions that occur, but only a very limited sequence ordering is possible. An inspection of 6,10,12 a-trihydroxy-1,3,11,12-tetraoxo-2,5,6,12a-tetrahydronaphthacene (TC1; m/z = 340) allows us to note that the following steps must have occurred, but in any number of different sequences: decarboxamidation at C2; deamination at C4; dehydroxylation at C5, demethylation at C6, and tautomerization at C3 and C12 [45] (Figure 6).

Similarly, OTC was converted to 3,4,4a,10-tetrahydro-1 2,8,10-tetrahydroxy-10-methyl-9-(2H)-anthracenone (TC2; m/z = 277) by ring-cleavage at both the C4aC4 and C12a-C1 bonds [46]. This step occurred either before or after dihydroxylation at C5. The observation of degradation intermediates TC1 and TC2 supports the idea of multiple oxidative pathways because TC2 cannot be produced by the degradation of TC1. This is because the methyl group at C6 in TC2 has already been removed in TC1. Finally, either of these compounds or many others could lead to the production of 2,3-dioxosuccinic acid by oxidative cleavage of any ring or sequence of carbon giving a four-carbon chain. Subsequent oxidation of each carbon of that chain would lead to succinic acid (m/z = 146) [47].

Further oxidation of that compound would probably lead to oxalic and carbonic acids, and then shortly thereafter to carbon dioxide and water. Thus, the oxidative fate of these compounds is likely to be complete mineralization under the reported conditions. Moreover, our LC/MS results also reveal that the adsorption process was the dominant process throughout the OTC reaction; however, the degradation products may be unstable and may have been adsorbed onto the catalyst surface.

### 3.5. Effect to Seedling Growth and Root Anatomy

**Figure 7:**
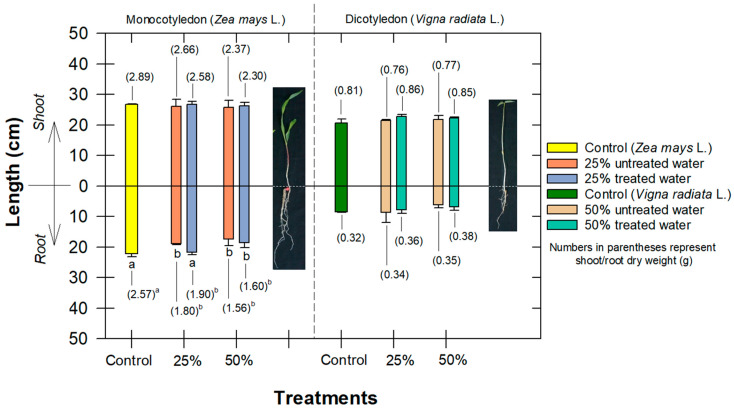
Comparison of shoot and root lengths and their dry weights (g; in parentheses) of *Zea mays* L. and *Vigna radiata* L. following exposure to different compositions of treated water. Values followed by the same letter in each species are not significantly different at *p* ≤ 0.05 according to the Tukey’s honest significant difference (HSD).

Up to a 50% composition of treated and untreated water, both species had no significant effect on shoot lengths or root lengths for *Vigna radiata* L. (Figure 7). This implies that all of the active radicals had already disappeared and that OTC degradates were not able to trigger *Vigna radiata* L. irregular plant growth nor cause any oxidative stress. This result is consistent with previous works indicating that *Vigna radiata* L. had an inherent ability for growth in chemically contaminated soils [48,49]. For *Zea mays* L. root traits, however, 50% of both tested waters revealed a significantly negative impact on the germination index, vigor index, and root length, while, at a lower composition of tested water (i.e., 25%), there were differences between untreated and treated water, indicating that our treated water was safer for root growth at this lower composition (Figure 7).

Water plays a crucial role in the germination process since it is necessary for the activation of enzymes that break down the seed’s stored nutrients and initiate the growth processes. Therefore, treated water at a high composition, which included OTC-degradates, a minimal amount of inorganic metal, and an abnormal pH, presumably initially interfered with the physiological and metabolic capacity of seeds to germinate and establish themselves as healthy seedlings. OTC-contaminated water at high concentrations has been confirmed to cause disruption in shoot and root growth by inhibiting the photosynthesis and enzyme activity of lettuce [50]. In addition, Bao et al. [51] showed that, when tested at higher concentrations of OTC (i.e., 50–150 mg L^−1^), the untreated OTC had significant phytotoxic effects on wheat seed germination, root elongation, sprout length, and vitality index. These investigations show the importance of removing as much antibiotic residues as possible before utilizing contaminated water for irrigation.

**Figure 8:**
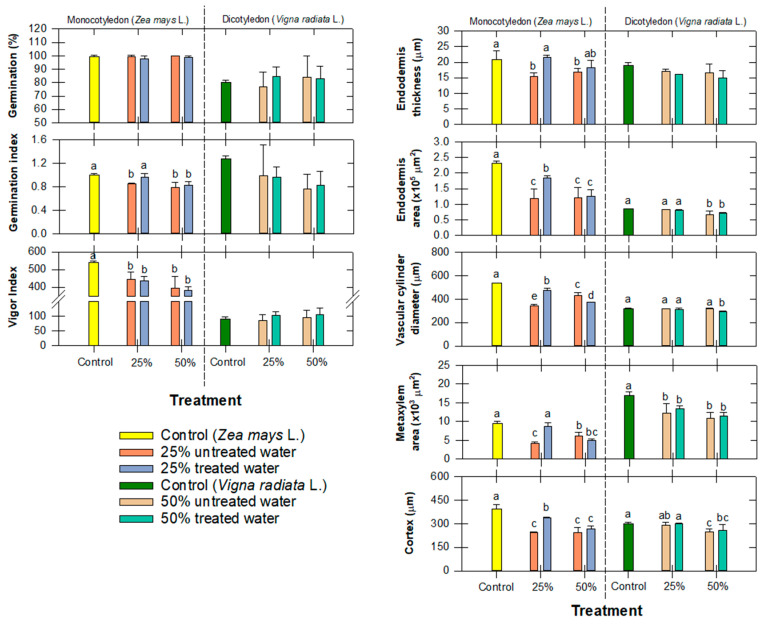
Comparison of seed germination, seedling growth, and anatomical roots of *Zea mays* L. and *Vigna radiata* L. following exposure to different compositions of treated water. Values followed by the same letter (s) in each species are not significantly different at *p* ≤ 0.05 according to the Tukey’s honest significant difference (HSD).

Following the seedling growth experiment, root samples were analyzed for changes in the anatomical parameters. Our findings reveal significant changes in root anatomy in response to the composition of treated water, where it showed a shrinking of the endodermis thickness and endodermis area, which could be due to the decrease in the size of the parenchyma of the cortex and vascular bundles (Figure 8).

For *Zea Mays* L., changes in endodermis area were observed at 50% of treated water, while changes in the vascular cylinder diameter, metaxylem area, and cortex were observed at only 25% of both treated and untreated water (Figure 8). However, the anatomical results for *Vigna radiata* L. are slightly different. In brief, only metaxylem and cortex were impacted by the tested water, but only 50% of the treated water started to negatively impact the endodermis area and vascular diameter, indicating that *Vigna radiata* L. could tolerate extreme conditions better than *Zea mays* L. (Figure 8). The *Vigna radiata* L. cortex obviously showed more negative impacts from the tested water. This is probably due to the nature of the larger root diameter (i.e., *Zea Mays* L.), which provided a longer radial distance containing more cortexes protecting the absorbed aqueous solution from penetrating into the endodermis area. At higher compositions of treated water, *Vigna radiata* L. root growth indicated that both OTC and OTC-degradates could disrupt maturation and cell division in the roots and may cause anomalous higher auxin production [52]. Because radical remnants were not the main by-products, we believed that either TC1 or TC2 (refer to Section 3.4; Figure 6) could be responsible for the reduction in parenchyma cells (Figure 8).

The reduction in root development is a compelling indication of plant oxidative stress, which results from a reduction in cell division, a decrease in cell elongation, and a reduction in the extension of the root meristem [53]. This decrease in cell size could also cause a reduction in cell wall elasticity, which can eventually lead to overall root contraction [54]. Gomes et al. [52] reported that this cell division impairment was due to the disruption of hormones that may have been associated with auxin production after exposure to treated water, and that any disturbances in auxin signaling can have profound effects on root anatomy and function.

Comparing the germination indices and the anatomical roots of both tested plants, it is clear that *Zea mays* L. was more sensitive than *Vigna radiata* L. Because of the root parameter reductions, it is plausible that the shoot would also suffer the same consequence. Because the plant’s root water potential was greater than its shoot water potential, the plant was able to absorb more water through its distorted xylem. Because of this, the plant’s capacity to absorb nutrients would have been compromised, and the OTC degradates would still be available for uptake by the roots. If the experiment had been set up for a longer time period (>8 d), we might have seen a different variation in shoot anatomy. In practical applications, a greater concentration of organic matter and other ion constituents can be found in water. Existing antibiotics may be present at much lower concentrations, and they would be naturally absorbed in soils before affecting root development. Most importantly, the dilution would be significantly lower than the mixture that was tested (<25%).

Past research has shown that a decrease in both cell division and cell size and a decrease in xylem vessel size are closely associated with the exposure to heavy-metal-contaminated water that causes the interference of the plant’s uptake of several mineral elements by heavy metals [55]. Although metal leaching was not an issue of concern in our study, some micronutrients were found to disrupt the plant mineral nutrient uptake, which consequently reduced enzyme activity, interfered with physiological processes, damaged cell membranes, and limited the biosynthesis of metabolites [56].

These changes in root anatomy can ultimately hinder the overall plant growth and development, can also highlight the sensitivity of root tissues to the composition of treated water, and can have cascading effects on nutrient uptake, water transport, and overall plant growth. Therefore, it is crucial to consider the potential impacts of treated water on root anatomy when assessing the suitability of water sources for irrigation and agricultural practices. Therefore, unless the amount of catalyst is increased to overcome the high loading of organic constituents, there should be no concern that these residuals will interfere with plant growth and yield. Therefore, our H_2_O_2_/Cu_0.5_Mn_0.5_Fe_2_O_4_ oxidative treatment was shown to be successful in removing antibiotics from water while having minimal to no negative effects on plant growth.

## 4. Conclusions

In this study, we developed Cu_0.5_Mn_0.5_Fe_2_O_4_ nanoparticles using the co-precipitation method, calcination with melamine, and tested their adsorptive–photocatalytic performance on four parabens and the oxytetracycline (OTC) removal efficiency. The material showed more crystallinity after melamine calcination and showed no Fe_2_O_3_ impurity, enhancing the larger adsorption sites as well as the electrons’ excellent oxygen exchangeability, which, in turn, can enhance oxidative reactivity for contaminant removal. All four parabens were removed within 120 min and OTC within 45 min, respectively. The optimum condition for pollutant removal was 0.2 g L^−1^ of Cu_0.5_Mn_0.5_Fe_2_O_4_ and 40 mM of H_2_O_2_. In the aqueous phase, both adsorptive and photocatalytic reactions can occur. The OTC diffused through the liquid phase and the film surrounding the adsorbent surface before interacting with the functional groups of the adsorbent–adsorbate. Then, also in the liquid phase, OTC rapidly reacted and generated reactive oxygen species. By fitting the kinetics and isotherm models, the OTC adsorption kinetics fitted well with the Brouers–Sotolongo model, and the OTC adsorption behavior was well described using the Freundlich isotherm. The shoot length of *Zea mays* L. and *Vigna radiata* L. in 25% treated water had less negative impact compared to the root length. The overall results show that Cu_0.5_Mn_0.5_Fe_2_O_4_ nanoparticles have provided a relatively good adsorptive–photocatalytic performance for the degradation of tested antibiotics and personal care products.

## Figures and Tables

**Table 1:** Kinetic and isotherm models used in this study.

Model	Equation	Ref.	Nomenclature
**Kinetic Model**			$A_{KC}$: Koble–Corrigan parameter ($L^{n_{KC}}{mg}^{1-n_{KC}}g^{–1}$) $A_{T}$: Temkin equilibrium binding parameter (L mol^–1^) $B_{DR}$: Dubinin-Radushkevich constant (mol^2^ J^–2^) $B_{KC}$: Koble–Corrigan parameter (${\left(L{mg}^{–1}\right)}^{n_{KC}}$) $B_{T}$: Temkin constant (J mol^–1^) b: Langmuir energy constant (L mg^–1^) $b_{K}$: Khan model constant (L mg^–1^) C: constant for intra-particle diffusion kinetic model (mg g^–1^) $C_{e}$: OTC concentration at equilibrium (mg L^–1^) $K_{BS}$: Brouers–Sotolongo isotherm constant (L mg^–1^) $K_{d}$: coefficient for intra–particle diffusion kinetic model (mg g^–1^ h^–1/2^) $K_{f}$: Freundlich constant (mg g^–1^(L mg^–1^)^1/n^) $K_{H}$: Hill constant ${\left(mgL^{-1}\right)}^{n_{H}}$ $K_{j}$: Jovanovich constant (L mg^–1^) $K_{RP}$: Redlich–Peterson isotherm constant (L g^–1^) $K_{T}$: Toth model constant (L mg^–1^) $k_{1}$: rate constant for pseudo first-order kinetic model (h^–1^) $k_{2}$: rate constant for pseudo second-order kinetic model (g mg^–1^ h^–1^) 1/n: Freundlich adsorption intensity $n_{KC}$: Koble–Corrigan parameter $n_{H}$: Hill cooperativity coefficient $n_{r}$: non-integer reaction order $n_{T}$: Toth model exponent $q_{e}$: amount of OTC adsorbed at equilibrium (mg g^–1^) $q_{m}$: maximum amount of the adsorbate per unit weight of the adsorbent (mg g^–1^) $q_{t}$: amount of OTC adsorbed at time t (mg g^–1^) R: universal gas constant (8.314 J K^–1^ mol^–1^) T: temperature (298 K) t: adsorption time (h) α: Elovich chemisorption rate (mg g^–1^ h^–1^) $\alpha_{bs}$: Brouers–Sotolongo model exponent $\alpha_{RP}$: Redlich–Peterson isotherm constant (${\left(L{mg}^{-1}\right)}^{\beta_{RP}}$) $\alpha_{K}$: Khan model exponent β: Elovich desorption rate constant (g mg^–1^) $\beta_{RP}$: Redlich–Peterson model exponent γ: fractal time exponent τ: characteristic time (h)
Pseudo first-order	$q_{t}=q_{e}\left(1-e^{-k_{1}t}\right)$	[18]	
Pseudo second-order	$q_{t}=\frac{q_{e}^{2}k_{2}t}{q_{e}k_{2}t+1}$	[18]	
Elovich	$q_{t}=\frac{1}{\beta }ln\left(\alpha \beta t\right)$	[18]	
Brouers- Sotolongo	$q_{t}=q_{e}\left(1-{\left(1+\left(n_{r}-1\right){\left(\frac{t}{\tau }\right)}^{\gamma }\right)}^{\frac{-1}{n_{r}-1}}\right)$	[19]	
Intra-particle diffusion	$q_{t}=K_{d}t^{1/2}+C$	[18]	
**Isotherm model**			
Langmuir	$q_{e}=\frac{q_{m}bC_{e}}{1+bC_{e}}$	[18]	
Freundlich	$q_{e}=K_{f}C_{e}^{1/n}$	[18]	
Temkin	$q_{e}=\frac{RT}{B_{T}}ln\left(A_{T}C_{e}\right)$	[18]	
Dubinin- Radushkevich	$q_{e}=q_{m}e^{-\left(B_{DR}\varepsilon^{2}\right)}$ $\varepsilon =RTln\left(1+\frac{1}{C_{e}}\right)$	[18]	
Jovanovic	$q_{e}=q_{m}\left(1-e^{-K_{j}C_{e}}\right)$	[18]	
Koble-Corrigan	$q_{e}=\frac{A_{KC}B_{KC}C_{e}^{n_{KC}}}{1+B_{KC}C_{e}^{n_{KC}}}$	[19]	
Khan	$q_{e}=\frac{q_{m}b_{K}C_{e}}{{\left(1+b_{K}C_{e}\right)}^{\alpha_{K}}}$	[19]	
Hill	$q_{e}=\frac{q_{m}C_{e}^{n_{H}}}{{K_{H}+C}_{e}^{n_{H}}}$	[19]	
Brouers- Sotolongo	$q_{e}=q_{m}\left(1-e^{-K_{BS}C_{e}^{\alpha_{bs}}}\right)$	[19]	
Toth	$q_{e}=\frac{q_{m}K_{T}C_{e}}{{\left(1+{\left(K_{T}C_{e}\right)}^{n_{T}}\right)}^{\frac{1}{n_{T}}}}$	[19]	
Redlich-Peterson	$q_{e}=\frac{K_{RP}C_{e}}{1+\alpha_{RP}C_{e}^{\beta_{RP}}}$	[19]	

**Table 2:** Parameters for the kinetic model determined from least squares approximation and model performance measured by RMSE and $R^{2}$.

Model	Cu_0.5_Mn_0.5_Fe_2_O_4_ (g)	Parameters				RMSE (mg/g)	$R^{2}$ (%)
Pseudo first-order		$q_{e}$ (mg g^−1^)		$k_{1}$ (h^−1^)			
	0.006	10.14		2.41		0.48	78.98
	0.012	5.94		5.03		0.07	83.92
	0.018	4.04		7.91		0.01	79.00
Pseudo second-order		$q_{e}$ (mg g^−1^)		$k_{2}$ (g mg^−1^ h^−1^)			
	0.006	10.82		0.38		0.25	94.50
	0.012	6.04		3.26		0.01	99.64
	0.018	4.06		20.72		<0.01	98.35
Elovich		α (mg g^−1^ h^−1^)		β (g mg^−1^)			
	0.006	1.45 × 10^3^		0.84		0.15	97.83
	0.012	6.79 × 10^12^		5.49		0.05	90.17
	0.018	2.78 × 10^53^		31.63		0.01	92.67
$\mathrm{Brouers}\text{–}\mathrm{Sotolongo}\;(\mathrm{order}\;2;\;n_{r}$= 2)		$q_{e}$ (mg g^−1^)	τ (h)		γ		
	0.006	14.07	0.33		0.38	0.15	97.92
	0.012	6.04	0.05		0.99	0.01	99.64
	0.018	4.07	4.50 × 10^−3^		0.78	<0.01	98.73
Intra-particle diffusion (two phases)		$K_{d}$ (mg g^−1^ h^−1/2^)		C (mg g^−1^)			
	0.006	10.54 if t≤0.58 h		0.00 if t≤0.58 h		0.07	99.52
		1.10 if t>0.58 h		8.03 if t>0.58 h			
	0.012	7.75 if t≤0.54 h		0.00 if t≤0.54 h		0.03	96.84
		0.13 if t>0.54 h		5.71 if t>0.54 h			
	0.018	5.61 if t≤0.51 h		0.00 if t≤0.51 h		<0.01	99.12
		0.02 if t>0.51 h		4.00 if t>0.51 h			

**Table 3:** Parameters for the isotherm model determined from least squares linear regression, strength of the linear relationship (between the independent and dependent variables in the linear form of the model) measured by $r^{2}$ and $r_{adj}^{2}$, and model performance measured by RMSE and $R^{2}$.

Model	Cu_0.5_Mn_0.5_Fe_2_O_4_ (g)	Parameters		$r^{2}$ (%)	$r_{adj}^{2}$ (%)	RMSE (mg/g)	$R^{2}$
Langmuir		$q_{m}$ (mg g^−1^)	b (L mg^−1^)				
	0.006	- *	- *	- *	- *	- *	- *
	0.012	994.94	1.08 × 10^−3^	98.82	98.63	63.08	77.42
	0.018	686.72	2.45 × 10^−3^	99.08	98.92	44.64	88.24
Freundlich		n	$K_{f}$ (mg g^−1^(L mg^−1^)^1/n^)				
	0.006	0.70	0.14	97.68	97.29	28.30	97.09
	0.012	0.93	0.74	99.06	98.90	14.65	98.78
	0.018	1.09	1.97	99.70	99.65	6.23	99.77
Temkin		$B_{T}$ (J mol^−1^)	$A_{T}$ (L mol^−1^)				
	0.006	14.68	14.47	82.87	80.01	68.60	82.87
	0.012	19.48	17.17	82.15	79.17	56.09	82.15
	0.018	19.98	21.34	86.60	84.37	47.66	86.60

* For Langmuir isotherm model applied to 0.006 g of Cu_0.5_Mn_0.5_Fe_2_O_4_, the values of parameters are out of appropriate range.

## Data Availability

The authors confirm that the data supporting the findings of this study are available within the article.
